# Relationship between gut microbiome diversity and hepatitis B viral load in patients with chronic hepatitis B

**DOI:** 10.1186/s13099-021-00461-1

**Published:** 2021-10-30

**Authors:** Eun-Jeong Joo, Hae Suk Cheong, Min-Jung Kwon, Won Sohn, Han-Na Kim, Yong Kyun Cho

**Affiliations:** 1 Division of Infectious Diseases, Department of Medicine, Kangbuk Samsung Hospital, Sungkyunkwan University, School of Medicine, Seoul, Republic of Korea; 2 Department of Laboratory Medicine, Kangbuk Samsung Hospital, Sungkyunkwan University School of Medicine, Seoul, Republic of Korea; 3 Division of Gastroenterology and Hepatology, Department of Medicine, Kangbuk Samsung Hospital, Sungkyunkwan University School of Medicine, Seoul, Republic of Korea; 4 Medical Research Institute, Kangbuk Samsung Hospital, Sungkyunkwan University School of Medicine, 29 Saemunan-ro, Jongno-gu, Seoul, 03181 Republic of Korea; 5 Department of Clinical Research Design & Evaluation, SAIHST, Sungkyunkwan University, Seoul, Republic of Korea; 6 Division of Gastroenterology and Hepatology, Department of Medicine, Kangbuk Samsung Hospital, Sungkyunkwan University School of Medicine, 29 Saemunan-ro, Jongno-gu, Seoul, 03181 Republic of Korea

**Keywords:** Hepatitis B, Chronic, Viral load, Gastrointestinal microbiome

## Abstract

**Background:**

Hepatitis B virus (HBV) infection is associated with a reduced risk of developing dyslipidemia and non-alcoholic fatty liver diseases. Given that the gut microbiota plays a significant role in cholesterol metabolism, we compared the differences in gut microbial diversity and composition between HBV-infected and uninfected subjects.

**Results:**

A prospective case–control study was designed comprising healthy controls (group A) and HBV-infected individuals (group B) in a 1:1 ratio (57 participants each; total = 114). The patients in group B were divided into two subgroups according to their HBV DNA loads: B1 < 2000 IU/mL (N = 40) and B2 ≥ 2000 IU/mL (N = 17). In a pairwise comparison of HBV-infected individuals and controls, higher alpha diversity was noted in group B, and the difference was significant only in patients in group B1. *Alloprevotella* and *Eubacterium coprostanoligenes* were predominant in group B1 compared to the control, whereas the abundance of *Bacteroides fragilis* and *Prevotella 2* was lower.

**Conclusions:**

The gut microbiome in HBV-infected individuals with a low viral load is highly diverse and is dominated by specific taxa involved in fatty acid and lipid metabolism. To our knowledge, this is the first demonstration of a correlation between the presence of certain bacterial taxa and chronic HBV infection depending on the load of HBV DNA.

**Supplementary Information:**

The online version contains supplementary material available at 10.1186/s13099-021-00461-1.

## Background

Chronic hepatitis B virus (HBV) infection is a notable health concern worldwide, especially in Asia and Africa, and is a major risk factor for fatality due to liver diseases such as cirrhosis and hepatocellular carcinoma (HCC). Although HBV is associated with several types of liver disease, previous cohort studies have provided significant evidence that HBV infections are linked to a reduction in serum cholesterol levels [1, 2], thereby reducing the risk of developing non-alcoholic fatty liver disease (NAFLD) [3]. HBV is considered a ‘metabolovirus,’ because the gene expression of HBV and the expression of key metabolic genes in hepatocytes were found to be similarly regulated [4, 5]. However, the underlying mechanisms of HBV-induced diseases currently unclear; therefore, no complete cure is available for chronic HBV infection [6]. A clearer understanding of the metabolic pathways involved in the pathological process of HBV infection may provide a new perspective on the potential novel targets of HBV treatment.

The gut microbiome, when considered a metabolic organ, has several roles in digestion, vitamin synthesis, immunomodulation, cardiovascular conditions, and the brain–gut axis. Consequently, it has an impact on the pathogenesis of gastrointestinal, hepatic, respiratory, cardiovascular, and endocrine diseases [7]. HBV infection has been found to alter dynamic changes in the gut microbiota [8, 9]. Given the significant role of the gut microbiota in cholesterol metabolism, it is possible that the effects of the altered gut microbiome in chronic HBV infection involve the regulation of microbe–host interactions at the gut interface. Here, we consider that the cholesterol-lowering properties of HBV infection may be attributable to the change of the gut microbiota by HBV infection. To evaluate the effect of the gut microbiome in chronic HBV infection on host metabolic health, we compared the differences in gut microbial diversity and composition between two groups of subjects: HBV-infected and uninfected subjects. Since gut microbiota is affected by different phases of disease in chronic hepatitis B and antiviral agents, we enrolled HBV-infected subjects in an inactive state who were not receiving antiviral treatment. We also investigated the differences in the gut microbiota with respect to viral loads among HBV-infected patients. A high viral load was defined as HBV DNA level ≥ 2000 IU/mL and a low viral load was defined as HBV DNA < 2000 IU/mL [10].

## Results

### Demographic characteristics of the study subjects

**Fig. 1:**
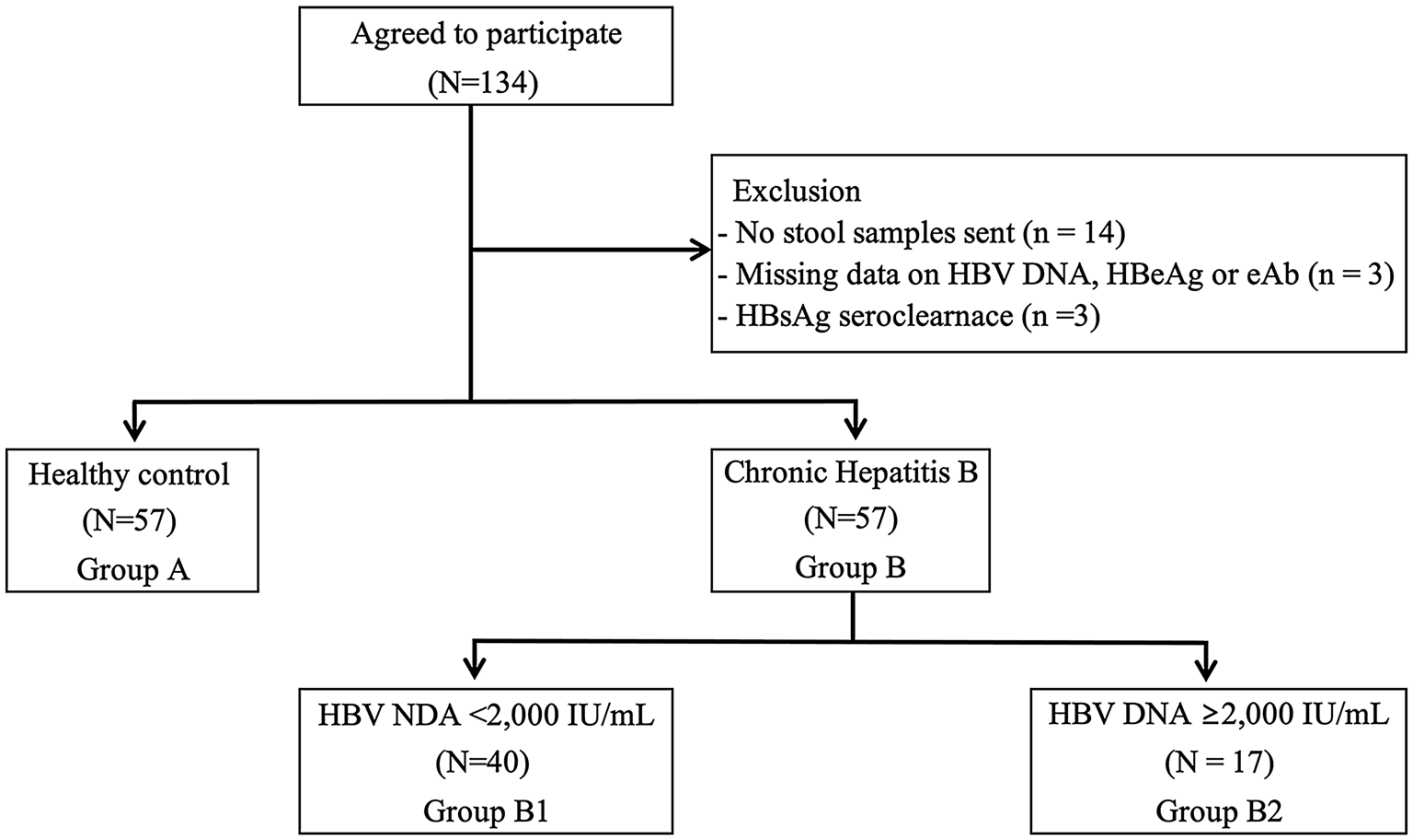
Enrolment of study participants

A total of 134 individuals participated in the study. Faecal samples were collected from 114 subjects, of which 57 (50%) were healthy subjects (controls, group A) and 57 (50%) were subjects with chronic HBV (group B). We excluded 20 patients from the initial group of participants; 14 of these had not sent in their stool samples; three had incomplete information on HBV DNA, hepatitis B e antigen (HBeAg), or hepatitis B e antibody (HBeAb); and three showed seroclearance of hepatitis B surface antigen (HBsAg) (Fig. 1). Group B was divided into two subgroups according to their HBV DNA loads: 40 patients were had a low viral load of HBV DNA (< 2000 IU/mL, group B1) and 17 had a high viral load of HBV DNA (≥ 2000 IU/mL, group B2).

The baseline characteristics of the study subjects are summarised in Table 1. The mean age and body mass index of the study participants were 45.59 and 23.21, respectively. Males accounted for 52.6% of the study participants. Compared with group A, old age, male sex, and current smoking were positively associated with group B. Laboratory findings showed that white blood cell and platelet counts were negatively associated and elevated hepatic enzymes were positively associated with group B compared to that in group A. There was a decreasing tendency in the low-density lipoprotein-cholesterol (LDL-C) and high-density lipoprotein-cholesterol (HDL-C) levels of those with HBV infection compared to group A. When comparing groups A and B1, similar trends were observed as when comparing groups A and B, except for the presence of lower HDL-C in group B1. Further subgroup analysis in group B showed that subjects in group B1 were associated with old age and low HDL-C, compared to those in group B2.

**Table 1:** Demographic and clinical characteristics of study participants

Characteristics	Healthy control	Chronic hepatitis B	*p* value^A, B^	HBV DNA IU/mL		*p* value^A, B1^	*p* value^A, B2^	*p* value^B1, B2^
				< 2000	≥ 2000			
	Group A	Group B		Group B1	Group B2			
Number	57	57		40	17			
Age (years)^*^	41.7 (7.91)	49.5 (9.64)	< 0.001	51.3 (9.01)	45.2 (9.9)	< 0.001	0.132	0.039
Male (%)	23 (40.4)	37 (64.9)	0.009	27 (67.5)	10 (58.8)	0.008	0.179	0.530
Current smoker (%)	1 (1.8)	8 (14)	< 0.001	4 (10%)	4 (23.5)	< 0.001	< 0.001	0.324
BMI (kg/m^2^)^*^	22.93 (3.11)	23.49 (3.05)	0.326	23.74 (2.96)	22.91 (3.27)	0.196	0.983	0.372
HBeAg (%)	–	6 (10.5)	–	0	6 (35.3)	–	–	< 0.001
HbeAb (%)	–	49 (86.0)	–	39 (97.5)	10 (58.8)	–	–	< 0.001
Previous antiviral agents (%)	–	3 (5.3)	–	0	3 (17.6)	–	–	0.023
Lab findings (mean, SD)								
WBC	6116 (1407)	5389 (1441)	0.007	5443 (1566)	5261 (1125)	0.029	0.025	0.667
Platelet	273,000 (59,060)	214,000 (51,740)	< 0.001	214,000 (54,330)	216,000 (46,600)	< 0.001	< 0.001	0.913
HbA1c	5.46^†^ (0.28)	5.46^†^ (0.38)	0.962	5.46^‡^ (0.33)	5.46 (0.51)	0.930	0.979	0.953
Total bilirubin	0.68 (0.25)	0.91 (0.39)	< 0.001	0.92 (0.39)	0.89 (0.40)	< 0.001	0.052	0.814
AST	21.18 (6.30)	23.86 (8.96)	0.067	22.98 (8.3)	25.94 (10.33)	0.228	0.086	0.304
ALT	22.93 (14.02)	27.51 (24.13)	0.218	25.85 (25.11)	31.41 ( 21.84)	0.466	0.146	0.407
Lipid profile (mean, SD)								
Total cholesterol	190.18 (34.14)	182.19 (32.64)	0.205	182.48 (32.62)	181.53 (33.67)	0.268	0.361	0.923
LDL-cholesterol	128.39 (33.83)	117.79^†^ (28.50)	0.075	120.46^‡^ (28.52)	111.65 (28.34)	0.233	0.068	0.294
HDL-cholesterol	63.28 (15.83)	57.41^†^ (17.35)	0.063	53.23^‡^ (13.83)	67.00 (20.97)	0.002	0.434	0.021

The χ^2^ test or Fisher’s exact test was used to compare groups A and B, A and B1, and B1 and B2

^*^Values are presented as mean (standard deviation)

^†^Values are presented as numbers of 56 and ^‡^values of 39

*SD* standard deviation, *LDL* low-density lipoprotein, *HDL* high-density lipoprotein

### Comparisons of gut microbial diversity among subjects with and without chronic HBV infections: alpha and beta diversity

We used 16S rRNA gene sequencing to determine the differences in gut microbiota composition according to HBV DNA load. The sequencing depth ranged from 21,459 to 102,066 reads per sample (mean = 47,752) and 1550 features in 114 subjects after contingency-based filtering of features.

**Fig. 2:**
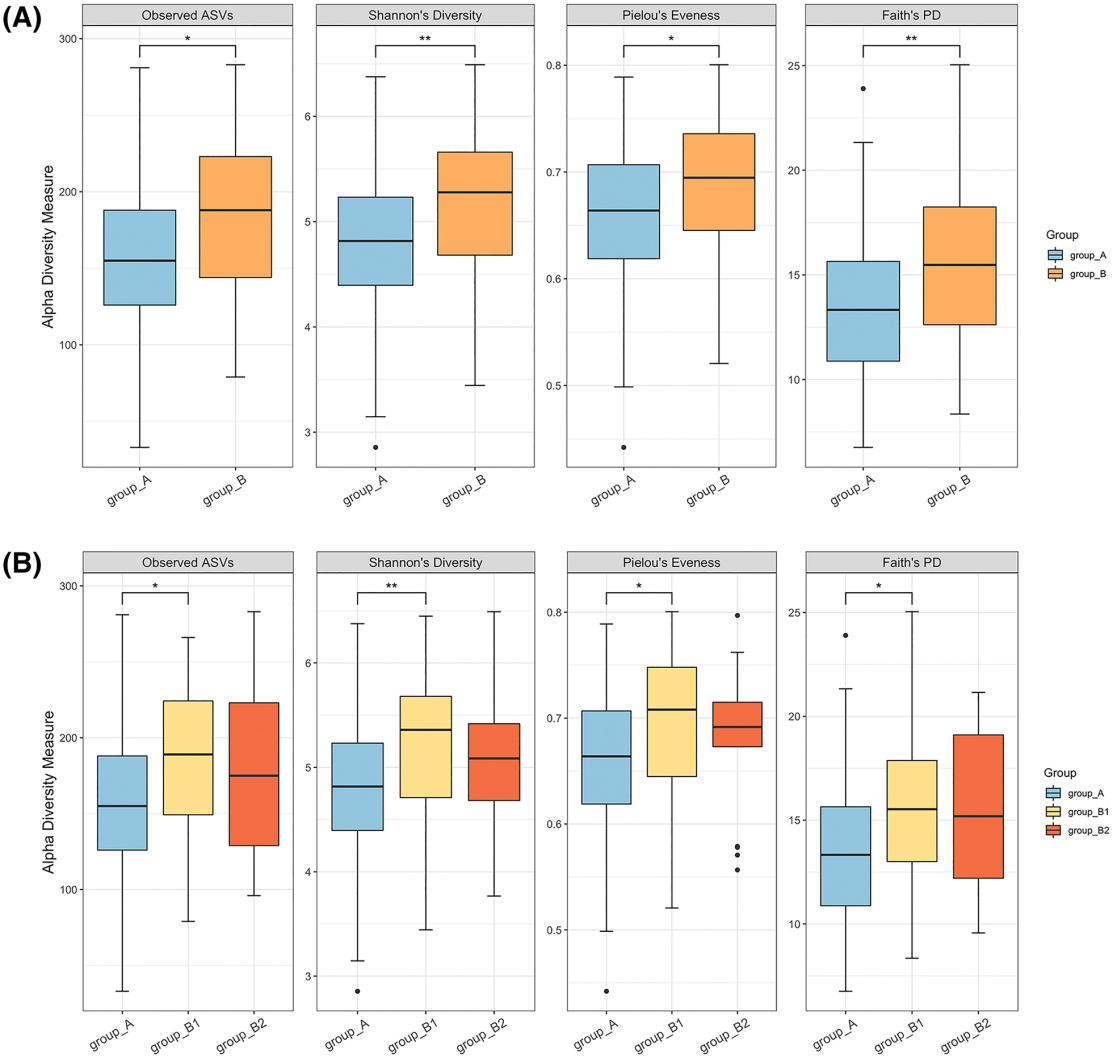
Alpha diversity. Comparison between subjects with and without HBV infection (**A**) and among three groups by hepatitis B viral load (**B**). ^*^q < 0.05, ^**^q < 0.01 (Mann–Whitney U test, Bonferroni correction). The boxes indicate the interquartile range. The median value is shown as a line within the box. Whiskers extend to the most extreme value within 1.5 × interquartile range

Group B showed significantly higher alpha diversity than group A in both the non-phylogenetic and phylogenetic indices (Fig. 2A and Additional file 1: Table S1). To investigate the extent of similarity and differences among microbial communities, we used both non-phylogenetic and phylogenetic indices for beta diversity analysis. Considering the presence or absence of the features, there were significant differences in the gut microbial community between groups A and B (*p* = 0.004 in Jaccard dissimilarity and *p* = 0.012 in unweighted UniFrac distance). However, no significant differences between groups A and B were observed in terms of the abundance of the features (Table 2).

**Table 2:** Statistical significance among groups by HBV DNA load using distance matrices for beta-diversity

	Group A vs. B		Group A vs. B1 vs. B2		Pairwise PERMANOVA					
Beta diversity Indices	pseudo-F	*p*	pseudo-F	*p*	*p* ^A, B1^	*q* ^A, B1^	*p* ^A, B2^	*q* ^A, B2^	*p* ^B1, B2^	*q* ^B1, B2^
Bray Curtis dissimilarity	1.255	0.148	1.090	0.250	0.365	1.095	0.174	0.522	0.471	1.413
Jaccard dissimilarity	1.593	0.004	1.258	0.019	0.01	0.03	0.112	0.336	0.574	1.722
Weighted UniFrac distance	0.625	0.595	0.478	0.884	0.784	2.352	0.586	1.758	0.863	2.589
Unweighted UniFrac distance	2.253	0.012	1.540	0.044	0.008	0.024	0.314	0.942	0.605	1.815

*q* values were calculated using Bonferroni correction

Pseudo-F statistics were calculated using pairwise PERMANOVA with 999 permutations

We then analysed the microbial diversity of each of the three groups: groups A, B1, and B2. The differences in alpha diversity among the three groups were mostly between groups A and B1, except for Pielou’s evenness (Fig. 2B and Additional file 1: Table S1). The pairwise test showed higher richness in group B1 than in group A (Bonferroni-corrected *q* = 0.039 in observed amplicon sequence variants (ASV) and *q* = 0.012 in Shannon’s index). Regarding phylogenetic diversity, Faith’s PD also revealed significantly higher diversity in group B1 than in group A (*q* = 0.039). When group B2 was compared with other groups, no statistically significant differences were observed in either non-phylogenetic or phylogenetic diversity. The beta-diversity analysis using permutational multivariate analysis of variance (PERMANOVA) revealed significant differences in microbial composition across the three groups in terms of Jaccard dissimilarity and unweighted UniFrac distance (*p* = 0.019 and *p* = 0.044, respectively; Table 2). The results of the pairwise PERMANOVA also demonstrated significant community differences between groups A and B1 (*q* = 0.030 in Jaccard dissimilarity and *q* = 0.024 in unweighted UniFrac distance), suggesting dissimilarities in the microbial composition of groups A and B1. However, owing to the high number of samples and inter-individual variation, the faecal microbiota in groups A and B1 could not be separated clearly by principal coordinate analysis (Additional file 1: Fig. S1); however, the differences in microbial community composition were significant between the two groups (Table 2). As in the results comparing groups A and B, the differences in bacterial communities among the three groups were also significant only in the indices of beta diversity in terms of the presence or absence of the features, whereas there was no significant difference in the indices regarding the abundance of features. Similar to the results of alpha diversity, the microbial community structure of group B2 was not significantly different from that of groups A or B1 (Table 2).

### Composition of gut microbiota among subjects with and without HBV infection

To identify the microbial taxa associated with HBV load, we performed association analysis using Analysis of composition of microbiomes (ANCOM). In total, 1550 ASVs were mapped to 1265 distinct taxa, including 14 phyla, 23 classes, 45 orders, 94 families, 326 genera, and 751 species. Bacteriodetes and Firmicutes were the most dominant phyla, comprising 48.4 and 46.6% of the total sequences, respectively; however, there were no significant differences in the levels of these phyla among the groups.

**Fig. 3:**
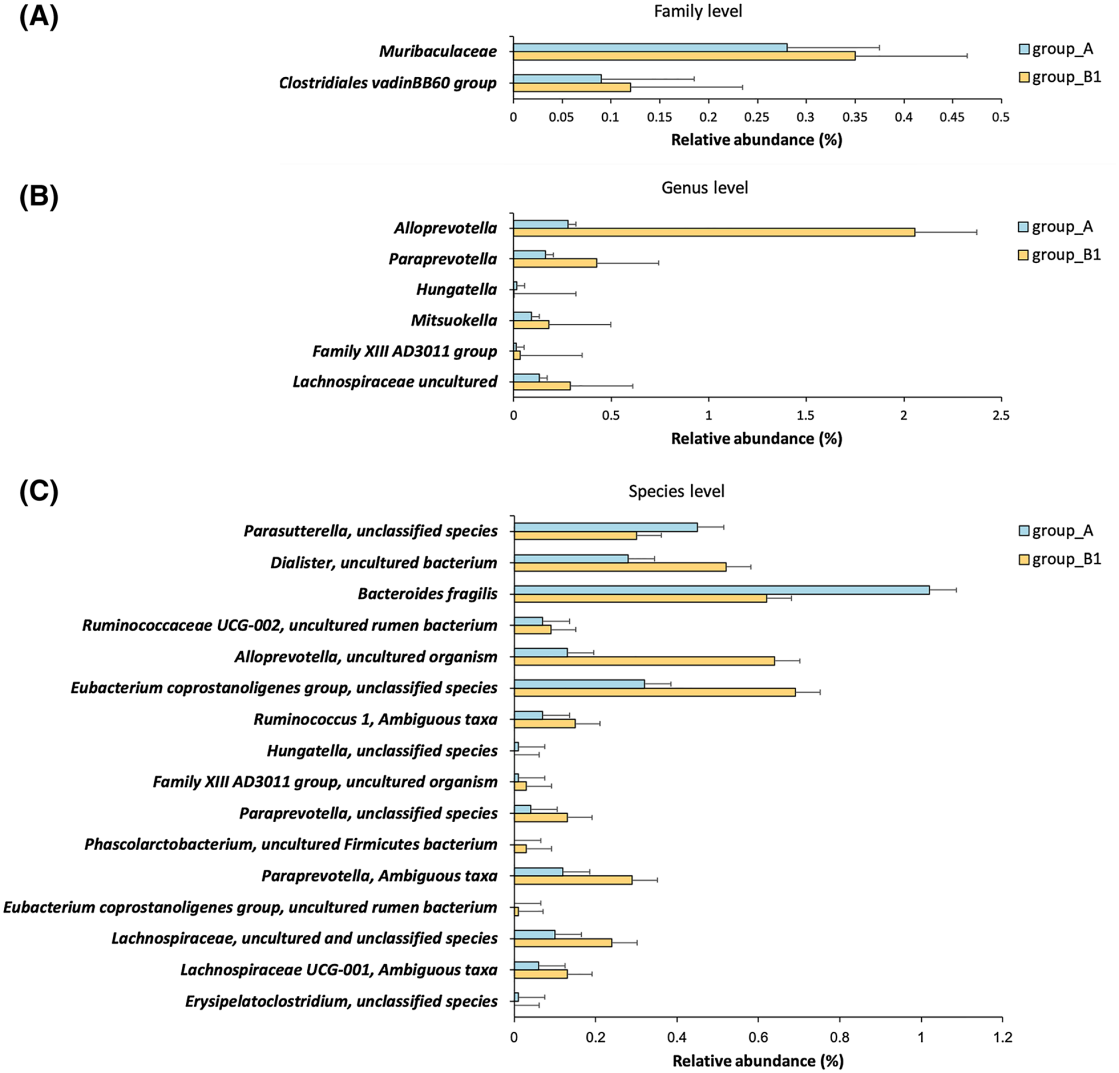
Relative abundance of taxa associated with HBV infection. Bar plots showing the relative abundance of family (**A**), genus (**B**), and species (**C**) levels in health controls (group A) and HBV-infected individuals with a low viral load (group B1)

We investigated the microbial composition of group A compared to that of group B. The ANCOM models showed several targets for HBV, including three genera (*Alloprevotella*, *Paraprevotella*, and *Hungatella*) and 17 species, after adjusting for age, sex, and smoking (Additional file 1: Table S2). We then compared the microbial compositions of groups A and B1 according to the results of the diversity analysis. There were no significant differences in the levels of phyla to order. However, significant differences between the two groups were found in two families, seven genera, and sixteen species (Additional file 1: Table S2). In Fig. 3, we present the relative abundances of the identified taxa. The genera *Alloprevotella*, *Paraprevotella*, *Hungatella*, *Mitsuokella*, and *Family XIII AD3011* and an uncultured genus of the family Lachnospiraceae showed significant differences compared to other genera when groups A and B1 were compared (*W* ≥ 230; ANCOM). The *W* statistics indicate that a taxon has significantly different compositions between groups when compared to over 70% of the total taxa, such as 230 genera of the total 326 genera. DADA2 resolved fine-scale variation better than the operational taxonomic unit clustering method and accurately reconstructed amplicon-sequenced communities at the highest resolution [11]; however, the amplicon sequencing of the 16S rRNA gene was best at the genus-level resolution [12]. Although we identified the taxa associated with HBV at the species level, most species with high *W* values were unclassified bacteria belonging to several genera such as *Alloprevotella*, *Paraprevotella*, *Family XIII AD3011*, *Ruminococcus*, *Hungatella*, and *Eubacterium coprostanoligenes* (Additional file 1: Table S2), except *for Bacteroides fragilis*. Further, when group B2 was compared with groups A or B1, no statistically significant taxon was found in the analysis of the relative abundance of bacterial taxonomic groups.

Based on the above results, we performed linear discriminant analysis effect size (LEfSe) to detect potential bacterial markers that most likely explain the differences between groups A and B1 by coupling standard tests for statistical significance with additional tests encoding biological consistency and effect relevance [13]. A comparison of groups A and B1 identified 16 taxa with linear discriminant analysis scores > 3 (Additional file 1: Fig. S2). The LEfSe results confirmed the significant enrichment of group *Eubacterium coprostanoligenes* in group B1 and found an enrichment of several species in *Alloprevotella* compared to the controls (group A). We also found that *Prevotella* 2 spp., *Hungatella* spp*.,* and *Bacteroides fragilis* were more abundant in the control than in group B1.

### Predicted functional microbiota in HBV-infected individuals with a low viral load (group B1)

**Fig. 4:**
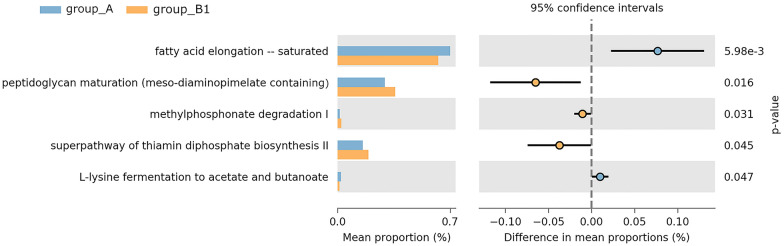
Predicted metabolic pathways that changed in group B1 compared with group A. Bar plots on the left side display the mean proportion of each pathway. Dot plots on the right show the differences in mean proportions between the two indicated groups using *p*-value. The *p*-values were not corrected with any multiple comparison method

To understand the gut microbial functions related to a low viral load (group B1), we used Phylogenetic Investigation of Communities by Reconstruction of Unobserved States 2 (PICRUSt2) to infer putative metagenomes from the 16S rRNA gene profiles. Statistical analysis of taxonomic and functional profiles (STAMP) was used to identify microbially relevant functions linked to HBV. However, after multiple comparison correction, we could not find any pathway that passed the significant threshold (Benjamini–Hochberg correction, FDR *q* < 0.05). Instead, Fig. 4 shows the suggested pathways with nominal significance (*p* < 0.05). The saturated fatty acid elongation pathway was lower in B1 than in A (*p* = 0.006), whereas peptidoglycan maturation, methylophosphonate degradation I, and the superpathway of thiamin diphosphate biosynthesis II pathways were enriched in B1 compared to A (*p* < 0.05).

## Discussion

In this prospective case–control study, we found that chronic HBV infection might be associated with a decreasing tendency in cholesterol levels, compared to the control group. A similar trend was observed in the groups of healthy controls and HBV-infected patients with a low HBV DNA level of < 2000 IU/mL. In a pairwise comparison between HBV-infected individuals and healthy controls, higher alpha diversity was noted in HBV carriers with a low viral load (group B1). Significant enrichment of *Alloprevotella* spp. and the *Eubacterium coprostanoligenes* was found in the low HBV DNA group subjects (group B1) compared to the controls (group A), whereas *Prevotella 2* spp., *Hungatella* spp*.,* and *Bacteroides fragilis* were more abundant in the controls than in group B1. This study demonstrated a correlation between the presence of certain bacterial taxa and chronic HBV infection according to the load of HBV DNA.

Gut microbiota dysbiosis was noted in HBV-infected individuals, with differential microbial communities evident in the healthy controls compared with each clinical stage of HBV infection such as chronic hepatitis B, cirrhosis, and HCC [14]. Based on previous findings in cohort studies that beneficial effects on lipid metabolism were identified in healthy HBsAg-positive individuals compared to controls [1, 2], the study participants in this study were those with HBV infection who had not received antiviral treatment and excluding cases of active hepatitis, cirrhosis, or HCC. It is of interest that HBV-infected patients with a low viral load of < 2000 IU/mL had a higher diversity in the gut microbiome compared to the healthy controls and patients with a high viral load of ≥ 2000 IU/mL. Patients with inactive chronic HBV infection, as indicated by HBeAg-negative, HbeAb-positive, persistently normal ALT, and a low viral load of < 2000 IU/mL, have an excellent long-term prognosis with low rates of histological progression, high rates of HbsAg clearance, and a very low risk of cirrhosis and HCC [15]. Considering that an inactive HBV carrier state is related to a better prognosis against progression to cirrhosis or HCC, it is notable that the gut microbiome in patients with HBV infection with low viral load has different properties, such as high diversity and microbial composition associated with lipid metabolism, suggesting that the gut microbiome in an inactive state of HBV infection may interplay between viral replication and the host immune and metabolic responses. Further investigations are needed to evaluate the relationship between viral factors and metabolic profiles in patients with chronic HBV.

*Alloprevotella* was positively associated with HBV-infected individuals with a low viral DNA load in comparison with healthy controls. In a previous study, *Alloprevotella* was negatively associated with lifetime cardiovascular disease risk, but *Prevotella 2* and *Prevotella 7* were positively associated with lifetime cardiovascular disease risk [16]. In another study, exposure to a high dose of diethyl phosphate enriched butyrate-producing genera such as *Alloprevotella*, leading to an increase in estradiol and a resulting decrease in triglycerides and LDL-C [17]. These bacteria are short-chain fatty acid producers and are negatively correlated with NAFLD [18]. Short chain fatty acids can be digested by the intestine and indirectly regulate energy metabolism and insulin sensitivity through specific receptors. Considering that *Alloprevotella* is associated with a reduced risk of cardiovascular disease and NAFLD, its predominance in HBV-infected subjects with a low viral DNA level might be associated with a decreased incidence of NAFLD in this population.

*Eubacterium coprostanoligenes* are anaerobic, non-sporing, gram-positive rod-shaped bacteria that reduce approximately 90% of the cholesterol in growth media to coprostanol [19, 20]. Unlike cholesterol, coprostanol is poorly absorbed by the human intestine, suggesting that it may have an impact on cholesterol metabolism and modulation of serum cholesterol levels [21]. It has been reported that most strains assigned to the genus *Eubacterium* exhibit cholesterol-reducing properties [22]. In this study, *Eubacterium coprostanoligenes* were predominantly found in HBV-infected individuals with low viral DNA loads, compared to the controls, which might mean that it is the main contributor to the cholesterol-lowering effects in HBV-infected individuals.

It is important to understand the role of the gut microbiome in the interactions between HBV infection and metabolic profiles. We found that the inferential pathway of saturated fatty acid elongation was enriched in non-hepatitis B individuals compared to that in hepatitis B-infected subjects with a low DNA load. There is evidence that long-chain saturated fatty acids activate certain transcription factors that target lipogenic target genes, sometimes leading to negative health effects. During type 2 diabetes progression, long-chain saturated fatty acids trigger cell apoptosis and increase oxidative stress [23]. Chronic exposure to long-chain saturated fatty acids is a risk factor for beta-cell apoptosis. The specific toxic effects of long-chain fatty acids may be related to ceramide formation and persistent endoplasmic reticulum stress. It is well established that excessive levels are contributory factors in the development of obesity, type 2 diabetes, and cardiovascular disease [24, 25]. These findings suggest that gut microbiota in inactive HBV carriers, which are characterized by less saturated fatty acid production, might play a major role in preventing hyperlipidemia compared to those in the controls.

Our study has several limitations. First, we excluded chronic hepatitis B patients who were receiving antiviral treatments such as nucleos(t)ide analogues because of active hepatitis with increased alanine aminotransferase (ALT) levels and high levels of HBV DNA. Antiviral treatment generally decreases HBV DNA to very low or undetectable levels. Therefore, a reduced viral level in patients undergoing antiviral therapy makes it difficult to assess the direct relationship between HBV DNA levels and the diversity of gut microbiomes. Second, although TG contributes to one component of metabolic syndrome, triglyceride levels were not measured in this study. Instead, we compared other lipid profiles, including total, LDL, and HDL cholesterol levels, among the study subgroups. Third, the sample size of group B2 was small compared to those of the other groups; therefore, we cannot exclude the possibility that the null results for group B2 might have been affected by the statistical power. Therefore, further investigation is warranted on the role of the gut microbiota in individuals with high HBV DNA load. Finally, the current study used 16S methods which cannot provide the taxonomic resolution at strain and information on several bacterial genes. Whole metagenomic sequencing might allow a wider range of understanding related to definitive taxonomic classification and functional profiling.

## Conclusions

The gut microbiome in HBV-infected individuals with a low viral load is highly diverse and is dominated by specific taxa involved in fatty acid and lipid metabolism*.* To the best of our knowledge, this is the first demonstration of a correlation between the presence of certain bacterial taxa and chronic HBV infection according to the load of HBV DNA.

## Methods

### Study participants and study design

The study included participants aged 18 years or older with at least one follow-up visit, who underwent serologic testing for HBsAg and lipid profiles, including total cholesterol, LDL-C, and HDL-C levels, during the study period. A prospective case–control study was designed with participants in a 1:1 ratio: healthy controls (*N* = 57) who underwent comprehensive health check-ups and patients (*N* = 57) with chronic HBV infection who visited the outpatient department of Gastroenterology and Hepatology at Kangbuk Samsung Hospital. Faecal samples were collected from the study participants for 7 months between 1 November 2018 and 31 May 2019.

We excluded subjects with previous or current use of antiviral agents for HBV infection, antibiotics, probiotics, proton-pump inhibitors, or cholesterol-lowering medication, and those with diabetes mellitus. In the case of patients with chronic HBV infection, any subject with liver cirrhosis or cancer related to HBV infection was also excluded.

### Chronic HBV infection and group definition.

Chronic HBV infection was defined as HBsAg-positive for > 6 months. HBsAg-negative participants were defined as healthy controls. The healthy subjects (controls) were designated as group A, whereas the patients with HBV infection were designated as group B. In the subjects with chronic HBV infection (group B), additional laboratory tests were also performed to evaluate HBV DNA levels, HBeAg, and HBeAb. This study included HBV-infected subjects with ALT levels < 2 upper limit of normal (80 IU/mL). To further distinguish inactive carriers from reactivated patients, we used a HBV DNA level of 2000 IU/mL to categorise HBV-infected subjects into two subgroups according to the viral load: low viral load of HBV DNA < 2000 IU/mL (group B1) and high viral load of HBV DNA ≥ 2000 IU/mL (group B2) [26, 27].

This study was approved by the Institutional Review Board of Kangbuk Samsung Hospital (KBSMC 2018–07-027, registered in July 2018). All study participants provided written informed consent to participate in the study. The study was conducted in accordance with the ethical guidelines of the World Medical Association Declaration of Helsinki.

### Measurements

We collected data on the medical history, medication use, smoking habits, and sociodemographic characteristics of the study subjects. Body mass index was calculated as the weight in kilogrammes divided by the height in meters squared. A complete blood count was performed using a haematology analyser XE‐2100 (Sysmex, Kobe, Japan). Serum ALT, aspartate aminotransferase (AST), total cholesterol, LDL-C, and HDL-C levels were determined using an automated chemistry analyser (Cobas c702; Roche Diagnostics, Tokyo, Japan). Haemoglobin A1c (HbA1c) levels were measured using an immunoturbidimetry immunoassay (Cobas c513; Roche Diagnostics, Tokyo, Japan). HBeAg and HBeAb were determined using an electrochemiluminescence immunoassay (Cobas e801; Roche Diagnostics, Tokyo, Japan) based on the sandwich principle. HBV DNA was quantified via real-time PCR using the COBAS AmpliPrep-COBAS TaqMan HBV assay (Roche Molecular Systems, Basel, Switzerland).

### DNA extraction from faecal samples and sequencing of the 16S rRNA gene

The OMNIgene-GUT collection kit (OMR-200, DNA Genotek, Ottawa, Canada) was used to collect stool samples. DNA extraction from faecal samples was performed within 1 month of storage using the MOBio PowerSoil DNA Isolation Kit (MO BIO Laboratories, Carlsbad, CA, USA) according to the manufacturer’s instructions. Amplification and sequencing were performed to analyse the bacterial communities as described previously [28]. Genomic DNA was amplified using fusion primers targeting the V3 and V4 regions of the 16S rRNA gene. Samples were pooled for sequencing on the Illumina MiSeq platform (Illumina, San Diego, CA, USA) according to the manufacturer’s instructions [29]. The DADA2 [11] plugin of the QIIME2 package (version 2019.7, https://qiime2.org) [30] was used to perform sequence quality control and construct a feature table of ASVs. For taxonomic structure analysis, taxonomy was assigned to ASVs using a pre-trained naïve Bayes classifier and the q2-feature-classifier plugin with the database Silva 132 release in the QIIME2 package.

### 16S rRNA gene compositional analysis and statistical analysis

Basic statistical analyses were performed using RStudio version 0.98.983 (Boston, MA, USA). For diversity analysis, the feature table was rarefied to 21,459 sequences per sample by random subsampling in QIIME2. To evaluate alpha diversity, we computed the number of ASVs observed in each sample, Shannon’s index, Pielou’s evenness, and Faith’s phylogenetic diversity (PD). The Mann–Whitney U test was used to test the differences between pairwise groups. The Kruskal–Wallis test was used as a non-parametric statistical test to compare the three groups. To measure beta diversity, we used the UniFrac distance [31] to estimate the dissimilarity among group members by incorporating the phylogenetic distances between ASVs. The unweighted and weighted UniFrac distances were calculated for the presence/absence and abundance of ASVs, respectively. Non-phylogenetic methods were also used with Bray–Curtis dissimilarities and Jaccard dissimilarity for the presence/absence and abundance of ASVs, respectively. Pairwise PERMANOVA with 999 random permutations was used to test the significance between groups.

Analysis of the composition of the microbiome via the ANCOM test [32] was performed to determine whether there were significant differences in the relative abundance of any taxa among the groups across multiple taxonomic levels. The final significance was expressed in the empirical distribution of *W* from the analyses of the two groups. We also conducted LEfSe analysis to detect potential HPV-specific bacterial markers [13].

For the functional inferences of the microbial community, we conducted the PICRUSt2 (v2.2.0-b) [33] with ASVs according to a recent manual https://github.com/picrust/picrust2/wiki). PICRUSt2 predictions based on the following gene families are supported by enzyme classification numbers (EC numbers, as of 21 January 2016). We generated PICRUSt2 EC gene family and metabolic pathway database (Metacyc) abundance predictions [34]. The results were visualised using STAMP version 2.1.3 [35] and tested using Welch’s t-test for two groups. All predictions were adjusted by multiple testing correction (Benjamini–Hochberg correction, FDR *q-value* < 0.05).

## Supplementary Information

**Additional file 1: Table S1**. Alpha diversity among groups of A1, B1 and B2. **Table S2**. Detection of differentially abundant taxa in all HBV carriers and HBV carriers with low viral DNA level compared to healthy controls. **Fig. S1**. Principal coordinates analysis of beta-diversity indices. **Fig. S2.** Low HBV DNA group-specific biomarkers.

## Data Availability

The datasets used and/or analysed during the current study are available from the corresponding author on reasonable request.

## Abbreviations

HBV: Hepatitis B virus
HCC: Hepatocellular carcinoma
NAFLD: Non-alcoholic fatty liver disease
LDL-C: Low-density lipoprotein-cholesterol
HDL-C: High-density lipoprotein-cholesterol
HBeAg: Hepatitis B e antigen
HBeAb: Hepatitis B e antibody
HBsAg: Hepatitis B surface antigen
ASV: Amplicon sequence variants
PERMANOVA: Permutational multivariate analysis of variance
ANCOM: Analysis of composition of microbiomes
LEfSe: Linear discriminant analysis effect size
STAMP: Statistical analysis of taxonomic and functional profiles
FDR: False positive rate
ALT: Alanine aminotransferase
AST: Aspartate aminotransferase
HbA1c: Haemoglobin A1c
