# Supplementary material for: Relationship between gut microbiome diversity and hepatitis B viral load in patients with chronic hepatitis B
Source: Gut Pathog. 2021 Oct 30;13:65. doi: 10.1186/s13099-021-00461-1 (PMC8557478; doi:10.1186/s13099-021-00461-1)

**Supplementary information**

**Relationship between gut microbiome diversity and hepatitis B viral load in patients with chronic hepatitis B**

**Table S1** Alpha diversity among groups of A1, B1 and B2

**Table S2** Detection of differentially abundant taxa in all HBV carriers and HBV carriers with low viral DNA level compared to healthy controls

**Figure S1** Principal coordinates analysis of beta-diversity indices. (A) Jaccard dissimilarity and (B) unweighted UniFrac distance.

The faecal microbiota in health controls (groups A) and HBV-infected individuals with a low viral load (group B1) could not be separated clearly by principal coordinates analysis

**Figure S2:** Low HBV DNA group-specific biomarkers.

Differentially abundant bacterial taxa in health controls (group A) and HBV-infected individuals with a low viral load (group B1). A forest plot showing the LDA score (effect size) indicating significant differences in the bacterial taxa between the group A (red) and group B1 (green) (LDA score > 3.0; *p* < 0.05).

**Supplementary Table S1** Alpha diversity among groups of A1, B1 and B2

|  | **Group A vs. B^†^** | |  | **Group A vs. B1 vs B2^‡^** | |  | **Pairwise-test^†^** | | | | | | | |
| --- | --- | --- | --- | --- | --- | --- | --- | --- | --- | --- | --- | --- | --- | --- |
| **Alpha Diversity Indices** | **W** | ***p*** |  | **H** | ***P*** |  | ***p* ^A, B1^** | ***q* ^A, B1^** |  | ***p* ^A, B2^** | ***q* ^A, B2^** |  | ***p* ^B1, B2^** | ***q*  ^B1, B2^** |
| Observed ASVs | 1190 | 0.014 |  | 6.394 | 0.041 |  | 0.013 | 0.039 |  | 0.225 | 0.674 |  | 0.595 | 1.784 |
| Shannon's Diversity | 1117 | 0.004 |  | 8.774 | 0.012 |  | 0.004 | 0.012 |  | 0.150 | 0.450 |  | 0.473 | 1.418 |
| Pielou's Evenness | 1211 | 0.019 |  | 5.980 | 0.050 |  | 0.018 | 0.055 |  | 0.242 | 0.727 |  | 0.411 | 1.233 |
| Faith's PD | 1160 | 0.009 |  | 6.931 | 0.031 |  | 0.013 | 0.039 |  | 0.111 | 0.333 |  | 0.870 | 2.609 |

*q* values were calculated using Bonferroni correction

^†^Mann-Whitney U test

^‡^Kruskal-Wallis test

**Supplementary Table S2** Detection of differentially abundant taxa in all HBV carriers and HBV carriers with low viral DNA level compared to healthy controls

| **Level** | **Taxa** | **W** | |
| --- | --- | --- | --- |
|  |  | **A vs B** | **A vs B1** |
| Family | D_1__Bacteroidetes;D_2__Bacteroidia;D_3__Bacteroidales;D_4__Muribaculaceae |  | 74 |
|  | D_1__Firmicutes;D_2__Clostridia;D_3__Clostridiales;D_4__Clostridiales vadinBB60 group |  | 72 |
| Genus | D_1__Bacteroidetes;D_2__Bacteroidia;D_3__Bacteroidales;D_4__Prevotellaceae;D_5__*Alloprevotella* | 283 | 287 |
|  | D_1__Bacteroidetes;D_2__Bacteroidia;D_3__Bacteroidales;D_4__Prevotellaceae;D_5__*Paraprevotella* | 260 | 278 |
|  | D_1__Firmicutes;D_2__Clostridia;D_3__Clostridiales;D_4__Lachnospiraceae;D_5__*Hungatella* | 248 | 231 |
|  | D_1__Firmicutes;D_2__Negativicutes;D_3__Selenomonadales;D_4__Veillonellaceae;D_5__*Mitsuokella* |  | 238 |
|  | D_1__Firmicutes;D_2__Clostridia;D_3__Clostridiales;D_4__Family XIII;D_5__*Family XIII AD3011* group |  | 230 |
|  | D_1__Firmicutes;D_2__Clostridia;D_3__Clostridiales;D_4__Lachnospiraceae;D_5__uncultured |  | 274 |
| Species | D_1__Proteobacteria;D_2__Gammaproteobacteria;D_3__Betaproteobacteriales;D_4__Burkholderiaceae;D_5__*Parasutterella*;D_6__unclassified | 705 | 651 |
|  | D_1__Firmicutes;D_2__Negativicutes;D_3__Selenomonadales;D_4__Acidaminococcaceae;D_5__*Phascolarctobacterium*;D_6__uncultured organism | 703 |  |
|  | D_1__Firmicutes;D_2__Negativicutes;D_3__Selenomonadales;D_4__Veillonellaceae;D_5__*Dialister*;D_6__uncultured bacterium | 700 | 672 |
|  | D_1__Bacteroidetes;D_2__Bacteroidia;D_3__Bacteroidales;D_4__Bacteroidaceae;D_5__*Bacteroides*;D_6__*Bacteroides fragilis* | 686 | 688 |
|  | D_1__Bacteroidetes;D_2__Bacteroidia;D_3__Bacteroidales;D_4__Bacteroidaceae;D_5__*Bacteroides*;D_6__*Bacteroides coprocola* DSM 17136 | 676 |  |
|  | D_1__Firmicutes;D_2__Clostridia;D_3__Clostridiales;D_4__Ruminococcaceae;D_5__*Ruminococcaceae UCG-002*;D_6__uncultured rumen bacterium | 649 | 588 |
|  | D_1__Bacteroidetes;D_2__Bacteroidia;D_3__Bacteroidales;D_4__Prevotellaceae;D_5__*Alloprevotella*;D_6__uncultured organism | 647 | 645 |
|  | D_1__Firmicutes;D_2__Clostridia;D_3__Clostridiales;D_4__Ruminococcaceae;D_5__*Eubacterium coprostanoligenes* group;D_6_unclassified | 643 | 573 |
|  | D_1__Firmicutes;D_2__Clostridia;D_3__Clostridiales;D_4__Ruminococcaceae;D_5__*Ruminococcus 1*;D_6__Ambiguous taxa | 619 | 602 |
|  | D_1__Bacteroidetes;D_2__Bacteroidia;D_3__Bacteroidales;D_4__Bacteroidaceae;D_5__*Bacteroides*;D_6__*Bacteroides uniformis* | 618 |  |
|  | D_1__Firmicutes;D_2__Clostridia;D_3__Clostridiales;D_4__Lachnospiraceae;D_5__*Hungatella*;D_6__unclassified | 613 | 596 |
|  | D_1__Firmicutes;D_2__Clostridia;D_3__Clostridiales;D_4__Ruminococcaceae;D_5__*Ruminiclostridium 5*;D_6__uncultured organism | 593 |  |
|  | D_1__Firmicutes;D_2__Clostridia;D_3__Clostridiales;D_4__Family XIII;D_5__*Family XIII AD3011* group;D_6__uncultured organism | 587 | 628 |
|  | D_1__Bacteroidetes;D_2__Bacteroidia;D_3__Bacteroidales;D_4__Prevotellaceae;D_5__*Paraprevotella*;D_6__unclassified | 578 | 633 |
|  | D_1__Firmicutes;D_2__Negativicutes;D_3__Selenomonadales;D_4__Acidaminococcaceae;D_5__Phascolarctobacterium;D_6__uncultured Firmicutes bacterium | 568 | 539 |
|  | D_1__Bacteroidetes;D_2__Bacteroidia;D_3__Bacteroidales;D_4__Rikenellaceae;D_5__Alistipes;D_6__Alistipes sp. N15.MGS 157 | 557 |  |
|  | D_1__Bacteroidetes;D_2__Bacteroidia;D_3__Bacteroidales;D_4__Prevotellaceae;D_5__Paraprevotella;D_6__Ambiguous taxa | 535 | 615 |
|  | D_1__Firmicutes;D_2__Clostridia;D_3__Clostridiales;D_4__Ruminococcaceae;D_5__Eubacterium coprostanoligenes group;D_6__uncultured rumen bacterium |  | 544 |
|  | D_1__Firmicutes;D_2__Clostridia;D_3__Clostridiales;D_4__Lachnospiraceae;D_5__uncultured;D_6__unclassified |  | 651 |
|  | D_1__Firmicutes;D_2__Clostridia;D_3__Clostridiales;D_4__Lachnospiraceae;D_5__*Lachnospiraceae UCG-001*;D_6__Ambiguous taxa |  | 574 |
|  | D_1__Firmicutes;D_2__Erysipelotrichia;D_3__Erysipelotrichales;D_4__Erysipelotrichaceae;D_5__*Erysipelatoclostridium*;D_6__unclassified |  | 531 |

**Supplementary Fig. S1.** PCoA analysis of beta-diversity indices. (A) Jaccard dissimilarity and (B) unweighted UniFrac distance


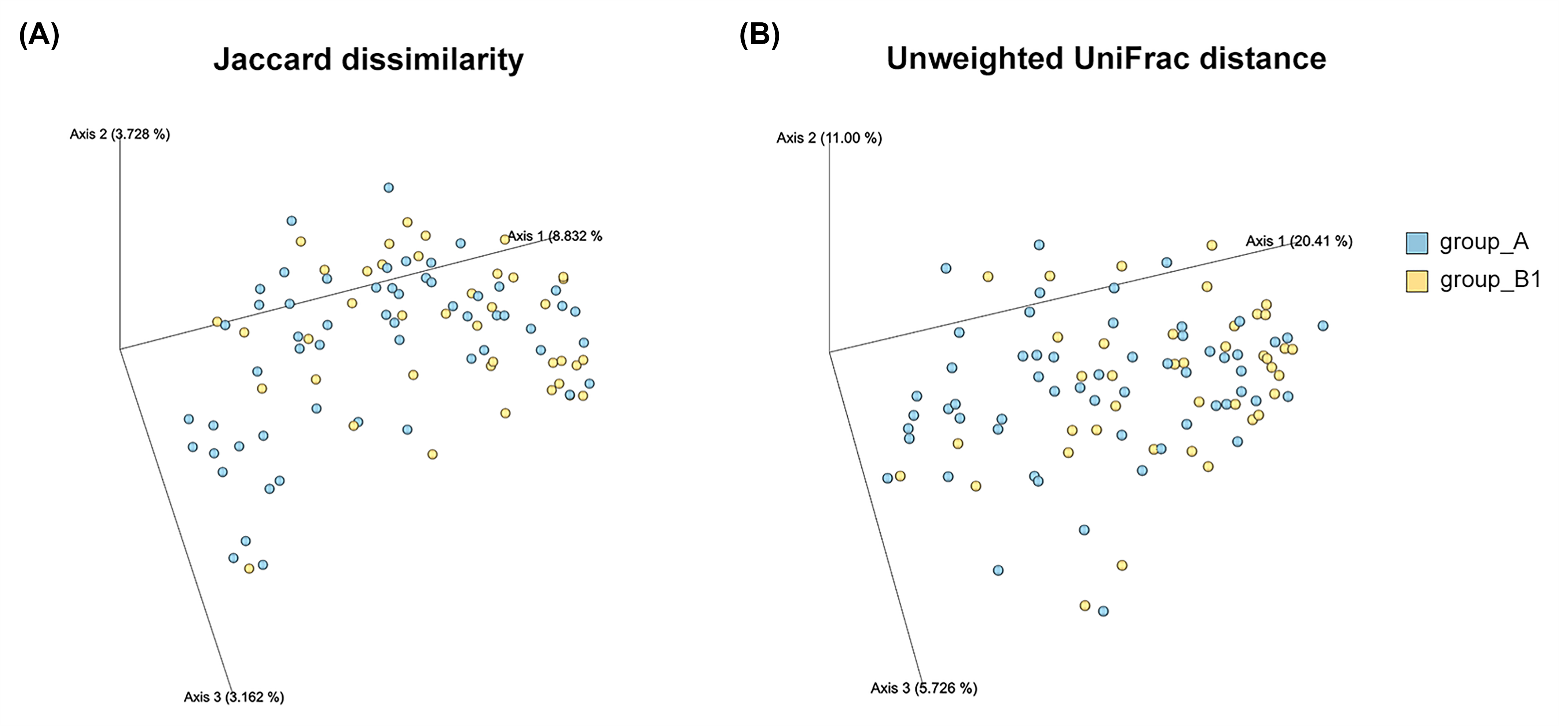


**Supplementary Fig. S2.** Low HBV DNA group-specific biomarkers.


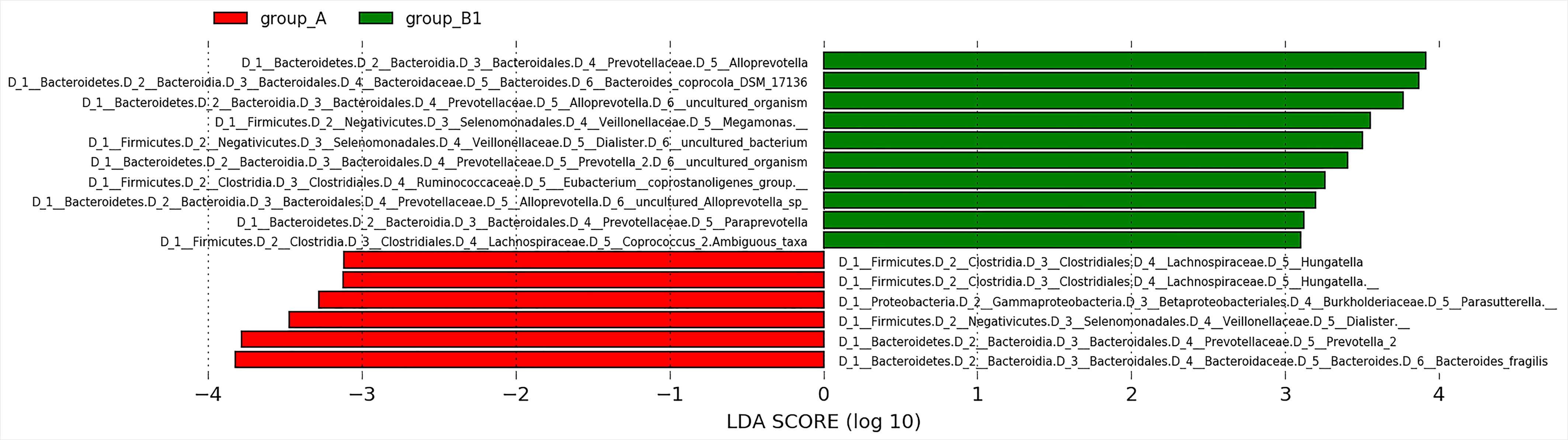

Supplement: Supplementary file 1 — Additional file 1: Table S1. Alpha diversity among groups of A1, B1 and B2. Table S2. Detection of differentially abundant taxa in all HBV carriers and HBV carriers with low viral DNA level compared to healthy controls. Fig. S1. Principal coordinates analysis of beta-diversity indices. Fig. S2. Low HBV DNA group-specific biomarkers. [file 13099_2021_461_MOESM1_ESM.docx]
